# *KRAS* and *NRAS* mutational gene profile of metastatic colorectal cancer patients in Jordan

**DOI:** 10.1371/journal.pone.0226473

**Published:** 2019-12-27

**Authors:** Muhammad Awidi, Nidaa Ababneh, Maha Shomaf, Feras Al Fararjeh, Laila Owaidi, Mohammad AlKhatib, Buthaina Al Tarawneh, Abdalla Awidi

**Affiliations:** 1 Beth Israel Lahey Health-Lahey Hospital and Medical Center, Burlington, Massachusetts, United States of America; 2 Cell Therapy Center, The University of Jordan, Amman, Jordan; 3 Department of Pathology and Microbiology and Forensic Medicine, The University of Jordan, Amman, Jordan; 4 Department of Medicine, The University of Jordan, School of Medicine, Amman, Jordan; 5 Hemostasis and Thrombosis Laboratory, School of Medicine, The University of Jordan, Amman, Jordan; 6 Department of Hematology and Oncology, Jordan University Hospital, Amman, Jordan; Ohio State University Wexner Medical Center, UNITED STATES

## Abstract

**Background:**

A constitutively active RAS protein in the absence of stimulation of the epidermal growth factor receptor (EGFR) is the result of mutations in *KRAS* and *NRAS* genes. Mutations in the *KRAS* exon 2 and outside exon 2 have been found to predict the resistance to anti-EGFR monoclonal therapy. A substantial proportion of metastatic colorectal cancer cases (mCRC) exhibit RAS mutations outside *KRAS* exon 2, particularly in *KRAS* exon 3 and 4 and *NRAS* exons 2 and 3. No data about RAS mutations outside *KRAS* exon 2 are available for Jordanian patients with mCRC. We aim to study the molecular spectrum, frequency, and distribution pattern of *KRAS* and *NRAS* mutations in Jordanian patients with mCRC.

**Methods:**

A cohort of 190 Jordanian metastatic colorectal cancer patients were enrolled in the trial. We detected mutations in exon 2 of the *KRAS* and *NRAS* gene as well as mutations outside of exon 2 using the StripAssay technique. The *KRAS* StripAssay covered 29 mutations and 22 *NRAS* mutations.

**Results:**

Mutations were observed in 92 (48.42%) cases, and *KRAS* exon 2 mutations accounted for 76 cases (83.69%). *KRAS* G12D was the most common mutation, occurring in 18 cases, followed by *KRAS* G12A in 16 cases, and G12T in 13 cases. Mutations outside of *KRAS* exon 2 represented 16.3% of the mutated cases. Among those, 6 cases (6.48%) carried mutations in *NRAS* exon 2 and 3, and 10 cases (10.87%) in *KRAS* exon 3 and 4.

**Conclusion:**

The frequency of *NRAS* and *KRAS* mutations outside of exon 2 appears to be higher in Jordanian patients in comparison with patients from western countries. *KRAS* mutations outside of exon 2 should be tested routinely to identify patients who should not be treated with anti-EGFR antibodies.

## Introduction

Colorectal cancer (CRC) is considered the most common type of cancer among males and the second most common type among females in the Jordanian population.[1] Recent significant advancements in the treatment of CRC have been achieved with new therapeutic approaches, which result from improved understanding of the molecular pathways involved in the development and progression of CRC.

Following ligand binding to the transmembrane receptor, the epidermal growth factor receptor (EGFR) forms a dimer that signals within the cell by activating the receptor auto-phosphorylation through its tyrosine kinase activity [2]. This intracellular signaling results in cancer-cell proliferation, enabling invasion, metastasis and stimulating tumor-induced neovascularization [2,3].

The v-Ki-Ras2 Kirsten rat sarcoma (*KRAS*) gene, first identified as an oncogene in the Kirsten rat sarcoma virus, is a member of the RAS gene family [4]; it is a downstream component of the EGFR signaling pathway [5]. *KRAS* acts as an intracellular signal transducer by coupling the signal from the cell surface receptor with different intracellular targets. Mutations in the RAS family are frequently found in many human tumors. Mutant RAS proteins are constitutively active in the absence of any upstream stimulation of the EGFR receptor [6]; this is due to the reduced intrinsic GTPase activity and insensitivity to GTPase activation proteins.

Mutations in the RAS gene occur in approximately 20% of all human cancers. [7,8] *KRAS* mutations account for about 85% of all RAS mutations in human cancers, while *NRAS* mutations account for about 15%. [9] In CRC, mutant *KRAS* is found in about 35–45% of cases [10,11]. Codon 12 and 13 on exon 2 of the *KRAS* gene are considered the two main 'hotspots,' together accounting for nearly 95% of all mutation types, with approximately 80% occurring in codon 12 and 15% in codon 13. Other mutations outside of exon 2 occurring in codon 61, 146 and 154 are less frequent in CRC and account for the remaining 5% of all mutation types. [12]

The anti-EGFR monoclonal antibodies cetuximab and panitumumab bind to the extracellular domain of EGFR when it is in the inactive configuration. The antibodies compete for the receptor binding by occluding the ligand-binding region, thereby blocking the ligand-induced EGFR tyrosine kinase activation.[3,13,14]

Mutations in the *KRAS* gene results in the continuous activation of signaling pathways without any upstream stimulation of the EGFR/HER receptors. [6] These mutations mediate the resistance to the anti-EGFR therapy, thus mandating RAS (*KRAS* exon 2, codon 12, 13) testing before the treatment with anti-EGFR therapy [15]. Recent studies revealed that despite having wild-type RAS, some patients with metastatic CRC (mCRC) had a reduced response to anti-EGFR therapy[16,17]. This would emphasize the importance of mutational analysis of *KRAS* exon 2, as well as outside exon 2 and *NRAS* gene. This mutational analysis should also be introduced as a routine screening test for mCRC patients who intend to receive cetuximab and panitumumab,to minimize drug toxicity and improve cost-effectiveness.[18]

In Jordan, patients with mCRC are routinely investigated for RAS mutations when considered for anti-EGFR therapy, but no data have been reported on *KRAS* mutations outside of exon 2. This work aimed to investigate the genotyping of *KRAS* mutations among Jordanian mCRC patients and to study the RAS mutations in exon 2 of *KRAS* and outside of exon 2.

## Materials and methods

### DNA extraction

DNA was extracted from paraffin-embedded tissue samples using QIAamp FFPE Tissue Kit (QIAGEN, Germany) according to the manufacturer instructions with few modifications. Briefly, 5–10 μm tissue sections were cut and washed in xylene for deparaffinization and then absolute ethanol (99%) solution was used to remove the paraffin. Samples were then centrifuged and the pellets were re-suspended in 180 μl ATL buffer, then treated with 20 μl proteinase K and incubated at 56°C for two hours. The lysed samples were then incubated at 90°C for one hour to reverse formaldehyde cross-linking. After a brief spin down, 200 μl AL buffer and 200 μl absolute ethanol (99%) were added directly to the samples and vortexed thoroughly. Samples were centrifuged, and 500 μl AW1 buffer was added and centrifuged. After that, DNA was eluted using 100μl ATE buffer and stored at -20°C for further use.

### *KRAS* and *NRAS* mutation analysis

The *KRAS* StripAssay (ViennaLab, Austria) covers 29 mutations in codons 12 & 13 (Exon 2), codons 59, 60 & 61 (Exon 3), and codons 117 & 146 (Exon 4). The *NRAS* StripAssay covers 22 mutations in codons 12 & 13 (Exon 2), codons 59, 60 & 61 (Exon 3), and codon 146 (Exon 4).

### PCR amplification and hybridization

Briefly, the *KRAS* gene sequence was amplified using a mixture of 15 μl amplification mix, 5 μl diluted Taq DNA polymerase (1U) and 5 μl DNA template (10 μg/ml). *KRAS* gene sequence was amplified using the following cycling conditions: initial incubation step at 37°C for 10 minutes and 94°C for 2 minutes, followed by 35 cycles of 94°C for 1 minute, 70°C for 50 seconds, 56°C for 50 seconds and 60°C for 1 minute, with a final extension step at 60°C for 3 minutes.

Finally, the amplification products were selectively hybridized to a test strip containing allele-specific oligonucleotide probes immobilized as an array of parallel lines. Bound biotinylated sequences were detected using streptavidin-alkaline phosphatase and color substrates. For each polymorphic position, one of the two possible patterns was obtained: either the presence of *KRAS* mutations hybridization bands or the absence of *KRAS* mutations.

## Results

In this study, 190 patient samples with metastatic colorectal cancer were analyzed for the presence of RAS gene mutations using Immunostrip technique. The median age at testing was 58 with a range of 19–83 years. Male patients accounted for more than half of the cases (n = 114, 60%). The mean age of female patients was slightly lower than that of male patients (55 ±12.36 vs. 58.48 ±12.31). The colon was the most likely primary tumor site (n = 182, 95.78%). There was a significantly higher frequency of left-sided colon cancer (n = 107, 56.32%) than right-sided (n = 62, 32.63%). Furthermore, the right-side tumors were more common in males (n = 37, 19.47%) compared to females (n = 25, 13.16%). The general characteristics of the patients tested are summarized in Table 1.

**Table 1:** Characteristics of the 190 patients.

**Gender, N (%)**	
Male	114, (60)
Female	76, (40)
**Median age at testing (range), years**	58, (19–83)
**Primary tumor site, N (%)**	
** Colon**	
Right	62, (32.63)
Left	107, (56.32)
Transverse	9, (4.74)
Sigmoid	2, (1.05)
NOS^a^	2, (1.05)
** Rectosigmoid**	3, (1.58)
** Rectum**	5 (2.63)
**Mutational Status, N(%)**	
Wild-type	98, (51.57)
Mutated	92, (48.42)

^a^ NOS: Not otherwise specified.

A total of 92 (48.42%) mutations in the RAS gene were identified. The *KRAS* mutations were described as mutations occurring in either exon 2 (n = 76, 83.69%) or outside of exon 2 (n = 16, 16.30%). Within the 76 cases of *KRAS* exon 2 mutations identified, the frequencies of mutation at codon 12, 13 and 117 were 81.57%, 17.1%, and 1.3% respectively. The majority of mutations occurred at codons 12 and 13 which accounted for more than 81% of the total mutated cases (98.68% of mutated cases on exon 2)

The glycine to aspartate on codon 12 (G12D) was the most common mutation, accounting for 18 (19.56%) of all the mutations identified. Mutations of glycine to alanine (G12A) was the second most common mutation (n = 16, 17.39%). While, mutation from glycine to threonine (G12T) and glycine to valine (G12V) constituted 13 and 10 (14.13% and 10.87%) of all mutated cases, respectively.

On the other hand, mutation from glycine to aspartate (G13D) was the most common mutation on codon 13 (n = 7, 7.60%) followed by mutation of glycine to alanine (G13A) (n = 6, 6.52%). One case demonstrated a lysine to asparagine (K117N) mutation on codon 117 (n = 1, 1.08%).

Ten different mutations were observed outside of *KRAS* exon 2; of those, four were *KRAS* mutations and six were *NRAS* mutations. The two most common *KRAS* mutations outside exon 2 were mutations from alanine to threonine on codon 146 (A146T) (n = 6, 6.52%) followed by lysine to asparagine (K117N) mutation on codon 117 (n = 2, 2.17%). Only one case harbored a *KRAS* mutation on codon 117 (K117N) and codon 146 (A146V) (n = 1, 1.08%) simultaneously.

*NRAS* testing was done if the *KRAS* mutation result was negative. Of the 104 samples analyzed by Immunostrip technique, six were positive for *NRAS* mutation (6.52%). All six mutations occurred once, with two located in exon 3, and the rest located in exon 2. A summary of all *KRAS* and *NRAS* mutations is shown in Table 2.

**Table 2:** Mutational status and detailed mutation classes found.

**Mutational Status**	N = 92, (%)
KRAS exon 2	76, (83.69)
Outside of KRAS exon 2	16, (16.3)
**KRAS exon 2**	N, (% of total mutated cases)
KRAS, exon 2, codon 12, G12D	18, (19.56)
KRAS, exon 2, codon 12, G12A	16, (17.39)
KRAS, exon 2, codon 12, G12T	13, (14.13)
KRAS, exon 2, codon 12, G12V	10, (10.87)
KRAS, exon 2, codon 13, G13D	7, (7.60)
KRAS, exon 2, codon 13, G13A	6, (6.52)
KRAS, exon 2, codon 12, G12S	3, (3.26)
KRAS, exon 2, codon 12, G12C	2, (2.17)
KRAS, exon 2, codon 117, K117N	1, (1.08)
**Outside of KRAS exon 2**	N, (% of total mutated cases)
KRAS, exon 4, codon 146, A146T	6, (6.52)
KRAS, exon 4, codon 117, K117N	2, (2.17)
KRAS, exon 4, codon 117, K117N & KRAS, exon 4, codon 146, A146V	1, (1.08)
KRAS, exon 3, codon 61, Q61H	1, (1.08)
NRAS, exon 3, codon 61, Q61R	1, (1.08)
NRAS, exon 3, codon 61, Q61P	1, (1.08)
NRAS, exon 2, codon 13, G13A	1, (1.08)
NRAS, exon 2, codon 13, G12C	1, (1.08)
NRAS, exon 2, codon 61, A61T	1, (1.08)
NRAS, exon 2, codon 12, G12V	1, (1.08)

## Discussion

Cetuximab and panitumumab are monoclonal antibodies that work by blocking the EGFR receptor, thus inhibiting its downstream signaling pathway. Mutations in any component of this pathway can make the treatment with cetuximab and panitumumab ineffective. Tumors with mutations in the *KRAS* gene, commonly in codon 12 and 13 of exon 2 or outside of exon 2 are virtually insensitive to cetuximab and panitumumab.[11,19]

In this study, we analyzed *KRAS* mutations in patients with mCRC: those occurring in exon 2 and outside of exon 2. The incidence of *KRAS* mutations in our study was approximately 48%, which is in line with what has been reported in a previous study in Jordan and other previous studies [20] [21,22] The majority of mutations fell in the *KRAS* exon 2 and constituted about 84% of the mutated cases. The frequencies of mutations in codon 12 and 13 were approximately 82% and 17% respectively. This suggests that the frequency and spectrum of *KRAS* mutations in our study are similar to what is reported in other studies[23,24].

In a previously published study, a prospective-retrospective analysis was carried out to assess the efficacy and safety of panitumumab together with either oxaliplatin, fluorouracil, or leucovorin (FOLFOX4), compared with FOLFOX4 alone and based on RAS (*KRAS* or *NRAS*) or *BRAF* mutational status. This study showed that patients with mutations in either *NRAS*, *BRAF*, or *KRAS* outside exon 2 genes had inferior progression-free survival (PFS) and overall survival (OS) with panitumumab-FOLFOX4 treatment.[17]

A systematic review and meta-analysis were carried out on nine randomized controlled trials to evaluate the anti-EGFR therapy on PFS and OS of tumors with *KRAS* exon 2 mutations compared to tumors without any RAS mutations and tumors with *KRAS* mutation in either exon 3 or 4 or an *NRAS* mutation in either exon 2, 3 or 4. The study demonstrated that tumors with no RAS mutations showed a significantly superior anti-EGFR PFS and OS treatment effect compared with tumors with a mutation in RAS genes. Further, no difference was observed in PFS and OS benefit between tumors with *KRAS* exon 2 mutations and tumors with *KRAS* exon 3 or 4 mutations or *NRAS* exon 2, 3 or 4 mutations. These results indicated that no PFS or OS benefit was obtained with the use of anti-EGFR therapy for tumors harboring any RAS mutation.[18]

The studies mentioned above also confirmed that although *NRAS* and *KRAS* outside of exon 2 mutations are less frequent than *KRAS* exon 2 mutations, they still predict the lack of response to cetuximab and panitumumab.

Of interest, *KRAS* exon 4, codon 146 (A146T) mutation was the most frequently detected mutation outside exon 2, constituting about 6% of the mutated cases. This is significantly higher than the frequency of the same mutation reported in the literature[16,25]. The *KRAS* mutations on codon 146 have been described in human colorectal cell lines[26]. Two studies from Hong Kong and the US detected codon 146 mutations in 9 out of 220 cases, giving a combined frequency of 4% [23]. The second most common mutation observed in our study, outside exon 2, was *KRAS* exon 4 codon 117 (K117N) mutation. This mutation constituted about a 2% mutation rate, which was significantly higher than what was reported in the literature[27].

*NRAS* mutations are considered rare in CRC, with one study detecting *NRAS* mutations in 2.2% of the 225 colorectal cancer cases.[28]. In our research, we found that *NRAS* mutations constituted approximately 6% (6/92) of all mutated cases.

The clinical significance of the *KRAS* mutations, except those of codons 12 and 13, remains unclear. *Loupakis et al*. reported that a patient with mCRC and *KRAS* 146 mutation was resistant to cetuximab[29]. In another study, they found that *NRAS* mutation carriers showed a significantly lower response rate than patients with wild-type *KRAS* when treated with cetuximab. [16]

This study suggests that Jordanian patients with metastatic colorectal cancer have a higher rate of *KRAS* outside exon 2 and *NRAS* mutations when compared to the literature.

Additional research with more samples is needed to study the effect of non-exon 2 mutations on the therapeutic outcomes.

## Conclusion

In summary, widening the *KRAS* mutational and subtyping analysis of colorectal cancer patients beyond the *KRAS* ‘hotspot’ codons 12 and 13 is useful in identifying patients who should not be treated with anti-EGFR antibodies, either alone or in combination with other anticancer agents. Because they are unlikely to benefit, and the exposure to toxicity and expense cannot be justified. A well designed prospective study to determine the full therapeutic implication of *NRAS* and *KRAS* outside of exon 2 mutation and validate the observational data is needed.

## Supporting information

S1 Dataset (XLSX)
